# Emergency Medical Management of Aneurysmal Subarachnoid Hemorrhage

**DOI:** 10.1007/s12028-023-01757-7

**Published:** 2023-06-21

**Authors:** Mervyn D. I. Vergouwen, Gabriel J. E. Rinkel

**Affiliations:** Department of Neurology and Neurosurgery, UMC Utrecht Brain Center, University Medical Center Utrecht, Utrecht University, Utrecht, The Netherlands

**Keywords:** Subarachnoid hemorrhage, Emergency care, Cerebral edema, Blood pressure hydrocephalus, Seizures, Hyperglycemia, Pulmonary edema, Takotsubo cardiomyopathy, Antifibrinolytics

## Abstract

Aneurysmal subarachnoid hemorrhage is a medical emergency that necessitates direct transfer to a tertiary referral center specialized in the diagnosis and treatment of this condition. The initial hours after aneurysmal rupture are critical for patients with aneurysmal subarachnoid hemorrhage, both in terms of rebleeding and combating the effect of early brain injury. No good treatment options are available to reduce the risk of rebleeding before aneurysm occlusion. Lowering the blood pressure may reduce the risk of rebleeding but carries a risk of inducing delayed cerebral ischemia or aggravating the consequences of early brain injury. Early brain injury after aneurysmal rupture has an important effect on final clinical outcome. Proper cerebral perfusion is pivotal in these initial hours after aneurysmal rupture but threatened by complications such as neurogenic pulmonary edema and cardiac stunning, or by acute hydrocephalus, which may necessitate early drainage of cerebrospinal fluid.

## Introduction

Aneurysmal subarachnoid hemorrhage (aSAH) is a medical emergency that necessitates direct transfer to a tertiary referral center specialized in the diagnosis and treatment of this condition. Twelve percent of patients die immediately before reaching the hospital [1, 2]. On admission to an emergency department, the priority in those with a decreased level of consciousness is to ensure adequate oxygenation and intubate if needed. In addition, the causes of a poor clinical condition should be determined and treated if possible, and patients should be admitted to an intensive or high care unit. After stabilization, medical care is focused on the prevention and treatment of complications to reduce the risk of death and poor functional and cognitive outcome.

The most important determinants of outcome are early brain injury, rebleeding of the ruptured aneurysm, and delayed cerebral ischemia (DCI) [3, 4]. Other common complications include hydrocephalus, electrolyte disturbances, infections, neurogenic pulmonary edema, stunned myocardium, and heart rhythm abnormalities. Despite current measures to prevent and treat SAH-related complications, admitted patients still have a 30% risk of 90-day case fatality, and many patients who survive have sequelae that hamper the return to work and social activities [4, 5]. In this review, we describe the medical management of patients with SAH within the first 24 h after ictus.

### Early Brain Injury and Acute Cerebral Edema

Early brain injury includes all processes leading to brain injury between the moment of aneurysm rupture and up to 72 h after ictus. Although early brain injury is the most important determinant of poor functional and cognitive outcome, it has attracted limited attention until recently. Most studies focused on the prevention and treatment of arterial narrowing (“vasospasm”) and DCI. Only after several studies showed that treatments that reduced the incidence of vasospasm did not improve functional outcome, focus started to shift from DCI toward early brain injury [6].

During rupture, blood spurs into the subarachnoid space under arterial pressure, leading to an acute rise in intracranial pressure. Blood in the ventricles and basal cisterns may lead to cerebrospinal fluid outflow obstruction and a further increase of intracranial pressure. As a result, the cerebral perfusion pressure decreases, and cerebral circulation may even become absent, which, depending on its severity and duration, may lead to global cerebral ischemia [7]. Cessation of cerebral blood flow may be transient, leading to a temporary loss of consciousness, long lasting leaving the patient comatose, or even permanent, leading to sudden death from aSAH. In patients who survive the initial episode of decreased cerebral circulation, the ischemic insult initiates secondary processes, such as microcirculatory disturbances and endothelial dysfunction with subsequent blood–brain barrier disruption and brain edema. Indeed, imaging studies have shown acute ischemic lesions and global cerebral edema, especially in patients with a poor clinical condition on admission [8, 9].

Currently, no treatment exists for early brain injury other than treatment of acute hydrocephalus and evacuation of an intracerebral or subdural hematoma to reduce intracranial pressure. Because cerebral edema is a common feature in patients with early brain injury, it may be tempting to consider treatment with steroids. Although the effect of steroids has been studied in a few small trials in patients with aSAH, the results were conflicting and steroid treatment was not focused on diminishing early cerebral edema but rather on the prevention or treatment of DCI or hyponatremia [10]. In conclusion, there is currently no evidence that treatment with steroids decreases the severity of early brain injury or improves functional or cognitive outcome after aSAH. The ongoing Fight INflammation to Improve outcome after aneurysmal Subarachnoid HEmorRhage (FINISHER)`trial aims to investigate whether treatment with dexamethasone as anti-inflammatory agent improves outcome after aSAH (144 of the intended 334 patients included on March 23, 2023) [11]. Another way to combat high intracranial pressure is hemicraniectomy. The PrImary decompressive Craniectomy in AneurySmal Subarachnoid hemOrrhage (PICASSO) trial investigates whether primary decompressive craniectomy performed within 24 h in addition to best medical treatment in patients with poor-grade SAH reduces case fatality and severe disability compared with best medical treatment alone and secondary craniectomy as a therapy of last resort for elevated intracranial pressure (58 of the intended 216 patients included on March 23, 2023) [12]. We see little value in intracranial pressure monitoring if evidence-based therapies that improve outcome by decreasing intracranial pressure from cerebral edema are lacking.

### Rebleeding of the Ruptured Aneurysm

A recently ruptured aneurysm has a 40% risk of rebleeding in the first 6 weeks after ictus, and rebleeding substantially increases the risk of poor outcome [13]. Although most instances of rebleeding occur within 24 h after ictus, available studies do not support that aneurysm treatment < 24 h results in better outcomes compared with treatment 24–72 h after the bleeding [14–16]. It has been hypothesized that the reduced risk of rebleeding in patients treated within 24 h is offset by an increased risk of cerebral ischemia. In addition, because most instances of early rebleeding occur within the first hours after the initial bleeding, it is difficult from a logistical point of view to prevent aneurysmal rebleeding before that time by endovascular or neurosurgical aneurysm treatment [17]. Therefore, medical (noninvasive) treatments to prevent aneurysm rebleeding are attractive. Below, we will discuss the effect of antifibrinolytic drugs and blood pressure control on rebleeding and functional outcome.

#### Antifibrinolytic Drugs

Shortly after aneurysm rupture, the tear in the aneurysm is sealed by a blood clot. The process of hemostasis depends on a balance between the hemostatic and fibrinolytic system. Especially in the early hours after aneurysm rupture, the blood clots that seals the tear in the aneurysm is unstable, which makes the aneurysm prone to recurrent bleeding. The rationale of treatment with antifibrinolytic drugs after aSAH is to improve clot stability and thereby decrease the risk of rebleeding. Several studies investigated the effect of antifibrinolytic drugs, such as tranexamic acid and epsilon-aminocaproic acid, on the incidence of rebleeding and functional outcome. Here, we will discuss the results of the major multicenter, randomized-controlled trials in this field [18–21].

In 1984, it was shown that long-term administration of tranexamic acid (given up to 4 weeks until aneurysm treatment) reduced the risk of rebleeding but did not improve functional outcome [18]. Although tranexamic acid reduced the incidence of rebleeding, there was no effect on functional outcome, which was explained by a higher incidence of ischemic complications. A new randomized trial was designed after oral nimodipine and maintenance of normovolemia became standard treatment to reduce the risk of DCI. The STAR trial, which was published in 2000, showed that long-term tranexamic acid reduced the risk of rebleeding without increasing the risk of DCI [19]. However, once again no effect on functional outcome was observed. Meanwhile, timing of aneurysm treatment started to shift from postponed treatment (often more than 14 days after ictus) toward treatment within 3 days. Therefore, a new randomized trial investigated the effect of short-term (< 72 h) treatment with tranexamic acid [20]. The results showed that short-term treatment reduced the incidence of rebleeding without increasing the risk of DCI and was associated with a trend toward improved functional outcome. Unfortunately, that trial was not powered to show any effect on functional outcome. More recently, the ULtra-early TRranexamic Acid after Subarachnoid Hemorrhage (ULTRA) trial investigated the effect of short-term (< 24 h) treatment with tranexamic acid with treatment initiation directly after computed tomography confirmed a diagnosis of SAH in the initial hospital and was powered to detect differences in functional outcome [21]. The median time between ictus and treatment initiation was 185 min. Rebleeding after randomization and before aneurysm treatment occurred in 49 (10%) patients in the tranexamic acid and in 66 (14%) patients in the control group (odds ratio 0.71, 95% confidence interval 0.48–1.04). Good functional outcome was observed in 287 (60%) of 475 patients in the tranexamic acid group and 300 (64%) of 470 patients in the control group (treatment center adjusted odds ratio 0.86, 95% confidence interval 0.66–1.12). The ULtra-early TRranexamic Acid after Subarachnoid Hemorrhage (ULTRA) trial provided convincing evidence that even very early and very short lasting antifibrinolytic therapy does not eliminate the risk of early rebleeding and does not improve functional outcome in patients with aSAH. An updated Cochrane review on this subject concluded that the current evidence does not support the routine use of antifibrinolytic drugs in patients with aSAH [22]. The recent Neurocritical Care Society guideline also recommends against the administration of antifibrinolytic therapy to prevent rebleeding of ruptured aneurysms in patients with aSAH [23].

#### Blood Pressure Control

Hypertension after aSAH is associated with rebleeding of the aneurysm, which can be explained by an increase in aneurysmal transmural pressure, and with poor functional outcome [24]. Although observations that treatment of hypertension may reduce the risk of rebleeding have existed for decades, there is no evidence from randomized trials that this treatment strategy improves outcome after aSAH. One of the first studies on blood pressure lowering that used computed tomography scans for diagnosis of clinical complications showed that blood pressure management (diastolic blood pressure target < 110 mm Hg) was associated with a lower incidence of rebleeding but a higher incidence of DCI [25]. A retrospective, single-center cohort study found a low incidence (3.1%) of rebleeding after the implementation of a strategy of intensive reduction of systolic blood pressure (target 120 ± 20 mm Hg) but did not include a control group [26]. Another retrospective, single-center study investigated the incidence of rebleeding before and after implementation of a blood pressure management protocol (systolic blood pressure target 160 mm Hg) [27]. After implementation of the protocol, a trend was observed toward a lower incidence of in-hospital rebleeding and a lower incidence of DCI compared with the time period prior to implementation of the protocol. A recent study compared the incidence of rebleeding between a hospital with a policy of aggressive blood pressure lowering versus one with a more liberal approach, and found a trend toward a lower incidence of rebleeding in the hospital with a more aggressive blood pressure lowering strategy [28]. However, there were differences in risk factors for rebleeding between the two centers, which introduced major bias.

Because all data on the value of blood pressure treatment in patients with aSAH come from low-quality studies, it is not surprising that the guidelines of the European Stroke Organization, American Heart/Stroke Organization, and Neurocritical Care Society have different recommendations [23, 29, 30]. The European Stroke Organization guidelines recommend a systolic blood pressure target < 180 mm Hg, starting with analgesics and nimodipine, and continuing as necessary to maintain a mean arterial pressure of > 90 mm Hg [29]. The American Heart/Stroke Association guidelines recommend blood pressure treatment with a systolic blood pressure target of < 160 mm Hg [30]. The Neurocritical Care Society guideline does not recommend a specific blood pressure target for the treatment of hypertension before aneurysm treatment in aSAH because of insufficient evidence [23].

A randomized clinical trial is required to answer the question of whether blood pressure lowering before aneurysm occlusion, and to which target, decreases the risk of rebleeding and improves outcome after aSAH. However, because strict blood pressure control does not eliminate the risk of rebleeding, several thousands of patients would need to be included in such a trial.

### Hydrocephalus

At least 20% of patients with aSAH have or develop hydrocephalus within the initial days after aneurysmal rupture. This so-called acute hydrocephalus is defined as a decreased level of consciousness combined with enlarged ventricles on imaging. The typical clinical picture is a gradual decrease in consciousness developing over hours. This deterioration in consciousness may be accompanied by small, fixed pupils and impaired upward gaze, although this latter sign cannot be investigated in patients with a severely depressed level of consciousness. These ocular signs may help in making the diagnosis of acute hydrocephalus, but absence of these signs does not exclude hydrocephalus as cause of the decreased level of consciousness. If acute hydrocephalus develops before the patient comes under medical attention, and the patient is already unconscious, the diagnosis can only be made based on enlarged ventricles on imaging. In individual patients with hydrocephalus as the only cause of a decreased level of consciousness, there is a good relation between the level of consciousness and the size of the ventricles, but at the group level the relation between level of consciousness and ventricular size is less clear.

Acute hydrocephalus may result from intraventricular obstruction through intraventricular extension of the hemorrhage, from compression of the ventricular system by an intracerebral hematoma or from obstruction of the flow of the cerebrospinal fluid at the level of the tentorial hiatus in patients in whom all cisterns around the brainstem are filled with blood [31].

The enlargement of the ventricles leads to increased intracranial pressure and reduced cerebral blood flow [32], and cerebrospinal fluid drainage to reduce the pressure can lead to a rapid improvement of the level of consciousness. The most often applied method is external ventricular drainage. If an external ventricular drain is placed before occlusion of the ruptured aneurysm, concerns have been raised regarding an increased risk of rerupture of the aneurysm, but in a study with controls matched for interval since aneurysmal rupture and duration of exposure, such a risk could not be confirmed [33]. Thus, the benefits of this procedure outweigh the risks, although large volumes of drainage should probably be avoided [34]. Intermittent drainage and rapid drain removal may help to reduce the risk of shunt dependency [35], but in a survey from 2017 this procedure was applied in less than 20% of the responding institutes [36]. In patients with hydrocephalus and severe intraventricular extension of the hemorrhage, intraventricular fibrinolysis after treatment of the ruptured aneurysm to resolve the clot and prevent drain obstruction has been found safe in preliminary randomized trials, but the effect on clinical outcome is unclear [37, 38]. In patients without intraventricular obstruction and obstruction of the cerebrospinal fluid flow at the level of the tentorial hiatus only, lumbar drainage (by needle puncture or catheter placement) is a reasonable and safe alternative [39]. In an uncontrolled series of 86 patients with acute hydrocephalus and a median Glasgow Coma Score of 10 (interquartile range 7–12) before lumbar puncture, 80% improved and 50% needed no external drainage [40].

### Seizures

Around one in every ten patients with aSAH has a seizure at time of aneurysmal rupture or shortly thereafter, but in many of these patients the clinical manifestation is more an epiphenomenon of a major neurological complication rather than an epileptic seizure [41]. Seizures at onset are more often seen in patients with poor clinical condition on admission, younger age, and aneurysms on the anterior circulation [41]. Accordingly, in patients with aSAH who have a seizure during transportation or early clinical course before occlusion of the aneurysm, an episode of rebleeding should always be suspected. Later on in the clinical course, again one of every ten patients develops seizures, but in those who had seizures at onset this risk can increase to one of every five patients [42]. Despite this 10% risk, there is no indication for routine prophylactic treatment with antiepileptic drugs. Besides lack of evidence of improved outcome after prophylactic treatment with antiseizure drugs, they may even lead to worse outcome [43, 44]. We reserve short time treatment with antiepileptic drugs to patients who have a first seizure not related to rebleeding during the clinical course. Currently, we see no role for continuous electroencephalography monitoring in patients with aSAH because there are no data suggesting that treatment with antiepileptic drugs in patients with electroencephalogram abnormalities improves outcome [45].

### Hyperglycemia

Hyperglycemia is found in one third of patients with aSAH during their clinical course and is associated with a poor clinical condition on admission [46]. The main contributors to hyperglycemia after aSAH are stress and inflammatory responses [47]. Both hyperglycemia on admission and hyperglycemia during the initial week after admission are independently associated with the occurrence of DCI [48] and with poor functional outcome, although a causal relationship remains uncertain [49]. Because of the association between hyperglycemia and DCI and poor outcome, several groups have studied the effect of glucose lowering therapy in patients with hyperglycemia after aSAH. In a systematic review of the literature from a decade ago, five cohort studies and one randomized trial investigating the value of intensive insulin therapy in patients with aSAH were identified. These studies showed no evidence that aggressively treating hyperglycemia in patients with aSAH was associated with beneficial effects, while conversely showing that intensive insulin therapy results in an increase in hypoglycemic episodes, raising serious concerns about its safety [47]. Similarly, in a recent randomized clinical trial including 78 patients, no effect of a 21-day course of daily 5-mg glibenclamide started within 96 h after ictus compared with placebo was found on clinical outcome at 6 months or on the occurrence of DCI [50].

### Neurogenic Pulmonary Edema

Pulmonary edema is a severe nonneurological complication after aSAH, which is most common in patients with a poor clinical condition on admission [51]. Pulmonary edema after aSAH can be cardiogenic (due to takotsubo cardiomyopathy) or neurogenic or a combination of both. The pathophysiology of neurogenic pulmonary edema is poorly understood. The acute rise in intracranial pressure probably leads to hyperactivity of the sympathetic nervous system, an increase of interstitial and alveolar fluid, and finally hypoxemic respiratory failure. Neurogenic pulmonary edema is a diagnosis of exclusion that requires ruling out left ventricular failure.

The management of neurogenic pulmonary edema after aSAH consists of supportive care and is focused on volume correction and avoidance of hypervolemia [51]. Hypovolemia should also be avoided because it is associated with the development of DCI. One study showed that a pulmonary artery catheter-guided hemodynamic management protocol reduces the incidence of neurogenic pulmonary edema after aSAH [52]. Supportive care must include adequate ventilation and sufficient oxygenation.

### Stunned Myocardium and Stress-Induced (Takotsubo) Cardiomyopathy

Stress-related cardiac abnormalities present with electrocardiogram changes, left ventricular dysfunction, and cardiac-specific serum enzyme and protein elevation. The characteristic finding on echocardiography is an aneurysm-shaped apex and hyperdynamic base, which resembles a takotsubo, a Japanese octopus (“tako”) trap (“tsubo”). In a systematic review of the literature including ten studies, one in every 20 patients with aSAH had a takotsubo cardiomyopathy [53]. Other distributions of wall motion abnormalities not related to the perfusion territory of a single coronary artery can also occur. In a prospective, multicenter, observational study including 301 patients who underwent serial echocardiography on admission and on days 4 and 8 after admission, 21% had wall motion abnormalities on admission and 16% had wall motion abnormalities on day 8 after admission [54]. The time course of the wall motion abnormalities was rather erratic; some patients had only wall motion abnormalities on admission, whereas others developed abnormalities during the clinical course, and other patients had wall motion abnormalities on two or all three echocardiograms. Poor neurological condition on admission was a risk factor for wall motion abnormalities on initial echocardiogram and increased troponin for development of wall motion abnormalities during the clinical course [54]. In the same cohort, the occurrence of wall motion abnormalities was an independent risk factor for poor outcome [55], which might relate to a decreased focal and global cerebral perfusion in those patients [56]. Because the wall motion abnormalities tend to resolve spontaneously, treatment consists of supportive management to prevent or minimize complications and is rather empiric because of the lack of solid evidence [57]. In our practice, we do not routinely obtain serum troponin assays or echocardiograms in every patient with aSAH, only if we suspect heart failure or the electrocardiogram is suggestive of myocardial ischemia.

### Current Management Considerations and Suggested Practice Guidance

The authors’ practice is as follows (Table 1):

- No antifibrinolytic drugs or corticosteroids.
- Upper blood pressure limits are based on individual factors. In general, we accept any systolic blood pressure after treatment of the aneurysm. In elderly patients, those with a history of cardiac disease, or those without hypertension prior to ictus, a blood pressure cutoff can be considered (for example systolic blood pressure below 220 mm Hg). If it is decided to give blood pressure lowering drugs, we prefer continuous infusion of short-acting drugs, such as labetalol. Diuretic drugs should be avoided to maintain normovolemia. Care should be taken that the blood pressure reduction will be gradual (maximum mean arterial pressure reduction of 10% in 2 h) to decrease the risk of DCI.
- Nimodipine 60 mg every 4 h.
- Antiepileptic drugs only after documented seizure is not related to (re)bleeding.
- Insulin therapy if fasting glucose > 10 mml/L, and regular glucose control with oral antidiabetic drugs and/or insulin therapy in patients with diabetes.
- Treatment of acute hydrocephalus with one or more lumbar punctures. We pursue external ventricular drainage only in case of complete filling of the third or fourth ventricle with blood if repeated lumbar punctures are not effective in restoring consciousness. External ventricular drainage is also applied if the neurosurgeon wants to decrease intracranial pressure prior to clipping of the ruptured aneurysm.

**Table 1:** Treatment options in emergency management of patients with aneurysmal subarachnoid hemorrhage

Type of treatment	Level of evidence	Authors practice
Prevention of rebleeding		
Antifibrinolytic drugs	Cochrane review and additional trials failed to show improved clinical outcome, despite reduction in rebleeding	Never
Blood pressure lowering	No trials available; guidelines recommend SBP < 160 (AHA), SBP < 180 (ESO), or no target (NCS)	Upper limits based on individual factors
Prevention of DCI		
Nimodipine	Cochrane review showed improved clinical outcome for oral (not intravenous) administration	Standard oral 60 mg every 4 h starting immediately after diagnosis
Corticosteroids	Two small RCTs found no beneficial effect on clinical outcome but an increased risk of hyperglycemia	Never
Prevention of seizures		
Antiepileptic drugs	No evidence for seizure reduction or improved outcome from prophylactic treatment; observational study suggests worse outcome after prophylactic treatment	Only after documented seizure unrelated to (re)bleeding
Glucose control		
Intensive insulin therapy	One trial and observational studies showed no benefit on outcome but increased risk of hypoglycemia	Insulin therapy if fasting glucose > 10 mmol/l; regular glucose control (oral diabetics and/or insulin therapy) in patients with diabetes
Glibenclamide	One small trial found no effect on DCI or clinical outcome	
Acute hydrocephalus		
Lumbar puncture	Only observational, noncontrolled case series	If treatment is indicated we start with one or more lumbar punctures, unless the third or fourth ventricles are entirely blocked by intraventricular blood; only in these patients and in patients in whom repeated lumbar punctures are not effective in restoring consciousness we pursue external ventricular drainage
External ventricular drainage	Only for risk of rebleeding retrospective controlled series. For clinical outcome noncontrolled series	

AHA, American Heart Association; DCI, delayed cerebral ischemia; ESO, European Stroke Organization; NCS, Neurocritical Care Society; RCT, randomized controlled trial; SBP, systolic blood pressure

### Gaps and Future Directions

- Identify novel treatments that improve recovery from early brain injury and thereby improve functional and cognitive outcome after aSAH.
- Determine the optimal blood pressure prior to aneurysm occlusion.
- Define the type and duration of cerebrospinal fluid drainage in patients with hydrocephalus.
- Determine the optimal treatment strategy for patients with takotsubo cardiomyopathy after aSAH.

## Conclusions

The initial hours after aneurysmal rupture are critical for patients with aSAH, both in terms of rebleeding and combating the effect of early brain injury. Unfortunately, no good treatment options are available to reduce the risk of rebleeding until the patient is admitted in a tertiary care center where the aneurysm can be occluded. Lowering the blood pressure may reduce the risk of rebleeding but carries a risk of inducing DCI or aggravating the consequences of early brain injury. The notion that early brain injury has an important effect on final clinical outcome has emerged over the last decade, but this process is, until now, poorly understood, and treatment strategies to reduce the effects of this injury need to be developed. Proper cerebral perfusion is pivotal in these initial hours after aneurysmal rupture but is threatened by complications such as neurogenic pulmonary edema and cardiac stunning, for which currently only supportive care is available. Cerebral perfusion can also be compromised by acute hydrocephalus, and early drainage of cerebrospinal fluid may improve perfusion and thereby final clinical outcome. The optimal type, duration, and extent of the drainage are uncertain, in particular before aneurysm occlusion. Supportive care to minimize secondary brain injury, prevention and treatment of cerebral and systemic complications, and initiation of oral nimodipine should be priorities in all patients presenting with aSAH.
